# Water depth-dependent stem elongation of completely submerged *Alternanthera philoxeroides* is mediated by intra-internodal growth variations

**DOI:** 10.3389/fpls.2024.1323547

**Published:** 2024-02-27

**Authors:** Shufang Jing, Xinyi Ren, Feng Lin, Hangang Niu, Qiaoli Ayi, Binna Wan, Bo Zeng, Xiaoping Zhang

**Affiliations:** ^1^ Key Laboratory of Eco-environments in Three Gorges Reservoir Region (Ministry of Education), Chongqing Key Laboratory of Plant Ecology and Resources in Three Gorges Reservoir Region, School of Life Sciences, Southwest University, Chongqing, China; ^2^ School of Biological Science and Food Engineering, Huanghuai University, Zhumadian, China

**Keywords:** alligator weed, cell proliferation, cell size, internode elongation, internode maturity, submergence depth

## Abstract

Complete submergence, especially deep submergence, poses a serious threat to the growth and survival of plants. One study previously showed that *Alternanthera philoxeroides* (a herbaceous perennial plant) submerged at depth of 2 m presented fast stem elongation and reduced stem elongation as water depth increased. In the present study, we aimed to figure out from the morphological and anatomical perspective how the differential growth response of the plant to water depth was achieved. We investigated the elongation of different stem parts and the relationship of stem elongation to cell size and number in *A. philoxeroides* by conducting experiments using a series of submergence depths (0 m, 2 m, 5 m, and 9 m). The results showed that, in comparison with unsubmerged plants, completely submerged plants exhibited enhanced elongation at depths of 2 m and 5 m but suppressed elongation at depth of 9 m in immature stem internodes, and displayed very little elongation in mature stem internodes at any depths. The stem growth of *A. philoxeroides* at any submergence depth was chiefly caused by the elongation of the basal parts of immature internodes. The elongation of the basal parts of immature internodes was highly correlated to both cell proliferation and cell enlargement, but the elongation of the middle and upper parts of immature internodes correlated nearly only with cell enlargement. This study provided new information on the growth responses of *A. philoxeroides* to heterogeneous submergence environments and deepened our understanding of the growth performance of terrestrial plants in habitats prone to deep floods.

## 1 Introduction

Submergence poses a serious threat to the growth and survival of terrestrial plants (Bejarano et al., 2018; Huang et al., 2021; Jing et al., 2022). Stress is exacerbated when a plant is completely submerged underwater, due to the shortage of carbon dioxide and oxygen because of their slow diffusion in water (Loreti et al., 2016). Plants growing in waterfront area of large reservoirs usually confront with submergence with large variation in depth owing to water level regulation. The Three Gorges reservoir, the largest hydroelectric power project in the world built on Yangtze River in China, has water level fluctuating regularly between 145 m (above sea level) and 175 m. Plants in the water level fluctuation zone of the reservoir have to experience submergence of 0~30 meters depth (Lei et al., 2014) which results in variable intensity of submergence stress. Although plants strive to cope with submergence in flood-prone habitats for their survival is a common phenomenon in natural world (Bashar et al., 2019), the knowledge of how plants respond and adapt to complete submergence of varying depths is still scarce.

Escape and quiescence strategies are two major strategies adopted by plants in flood-disturbed habitats (van Veen et al., 2014). In general, escape strategies enable plants to cope with shallow but long-duration submergence, whereas quiescence strategies favor plants to withstand deep and short-term submergence (Striker et al., 2017; Bashar et al., 2019). The “escape strategy” syndrome consists of rapid shoot or petiole elongation to rapidly regain contact with air so as to improve oxygen uptake under submergence conditions (Müller et al., 2019). Plants adopting quiescent strategies are more successful under deep submergence conditions (Manzur et al., 2009), they perform only basic metabolism with little or no organ elongation and new tissue formation, thereby suppressing energy expenditure, conserving carbohydrate reserves while waiting for the water level to drop, and prolonging survival in deep water (Manzur et al., 2009; Luo et al., 2011). Most plants use either an escape strategy or a quiescence strategy to cope with submergence. However, it is found that a special plant, *Alternanthera philoxeroides*, does not follow this either-or strategy selection but embrace both strategies by adopting an escape strategy in shallow flooded environments and a quiescence strategy upon deep submergence (Jing et al., 2022); it can gradually change its response strategy from “escape” to “quiescence” when water depth increased. Field works indicated that *A. philoxeroides* can live in the water level fluctuation zone possessing different submergence depths in the Three Gorges reservoir (Zheng et al., 2021), which may be very likely due to the capability of the plant to change strategies. While the formation of pith cavity and adventitious roots, and non-structural carbohydrate metabolism were found playing roles in the strategy change of *A. philoxeroides* along submergence depths (Jing et al., 2022), the detailed growth mechanism driving the change remains unclear.

When terrestrial plants are subjected to complete submergence, their aboveground parts undergo drastic changes in growth pattern, tissue structure, cell formation, and physiological process (Bailey-Serres and Voesenek, 2008; Müller et al., 2019; Jing et al., 2022). Moreover, the growth behavior of plants under submergence conditions are maturity-dependent (Groeneveld and Voesenek, 2003; Müller et al., 2019). For example, after being submerged for 10 days, the submergence-induced petiole elongation in *Rumex palustris* depends on the developmental stage of the petioles (Groeneveld and Voesenek, 2003). Very young *R. palustris* petioles had limited capacity to elongate, but slightly older petioles obtained the longest final leaf lengths (Groeneveld and Voesenek, 2003). In contrast, in deepwater rice, only the youngest internodes responded significantly to submergence (Sauter, 2000). Internodes of submerged deepwater rice can elongate by up to 20~25 cm in a period of days (Ayano et al., 2014), and the growth of the meristematic zone, elongation zone and maturation zone of young internodes in deepwater rice differed under submergence, suggesting that the effect of submergence on different plant parts is not the same. Our previous study showed that the growth response of *A. philoxeroides* to different submergence depths differed (Jing et al., 2022). However, it is unclear whether the growth response differs between internodes of different maturity.

Basically, the morphological response of plants to submergence is based on changes in cell number and cell size (Taiz and Zeiger, 2010; Crang et al., 2018). However, the contribution of cell number and cell size to plant growth remains controversial. It has been argued that it is cell size, rather than cell number, causes differences in plant growth, e.g., cell length instead of cell number is the predominant factor affecting hypocotyl length in *Medicago truncatula* (Youssef et al., 2016). When rice seeds germinate under complete submergence, increase in cell number determines leaf sheath elongation, which is significantly and positively correlated with cell division (Fang et al., 2018). In deepwater rice, the shoot can increase rapidly with increasing water depth, and shoot elongation mainly results from the cell division of intercalary meristem at the base of young internodes (Kende et al., 1998). Cell division and growth require cell wall expansion, cellulose microfibrils play essential roles in this process (Taiz and Zeiger, 2010). The microfibrillar network can be easily disturbed by external forces, causing abnormal microtubule depolymerisation and polymerisation, which affects cell division and elongation in plants (Jacques et al., 2013). As the depth of submergence increases, the effect of water depth on plant elongation growth can be easily observed, but how this effect comes into being, especially its underlying morphological and anatomical details, is still unknown.

To explore the morphological and anatomical mechanisms of plant growth responses to water depth, taking *Alternanthera philoxeroides* (Mart.) Griseb., a submergence-tolerant plant as a model (Jing et al., 2022), we conducted submergence experiments with several water depths (0 m, 2 m, 5 m, and 9 m) to answer the following questions: (1) Do the growth of mature and immature stems differ as submergence depth changes? (2) Can the different constituent parts within an immature internode achieve same elongation growth during submergence? (3) Do the cell size and cell number differ between constituent parts of an immature internode following submergence? (4) Do cell proliferation and cell enlargement within an internode play equally important roles in internode elongation? The answers to these questions will expand and deepen our understanding of how *A. philoxeroides* responds and adapts to flooding of varying depths, and provide insights into the contribution of internodes of different maturity and the contribution of internodal constituent parts to the elongation growth of plants upon submergence.

## 2 Materials and methods

### 2.1 Plant material and growth conditions

*Alternanthera philoxeroides* (Mart.) Griseb., a terrestrial perennial plant of Amaranthaceae, as described in Jing et al. (2022), was used in this study. *A. philoxeroides* plants were cultivated from cuttings obtained from plants naturally growing on the banks of the Jialing River in Chongqing, Southwest China (29°49’N, 106°25’E). Each selected cutting was planted in a plastic pot (diameter and depth were both 13 cm) containing riparian soil from the Jialing River banks. All plants were cultivated under the same conditions. The temperature, relative humidity, daily maximum light intensity, and water provision were maintained at 10~15°C, 75~85%, 600~800 µmol m^–2^ s^–1^, and approximately 80~90% of the soil water-holding capacity, respectively. After about one month cultivation, plants with 45 cm height approximately and 12 internodes were selected for the study.

### 2.2 Experimental design

Five groups of plants (20 replicate plants per group) were selected for the study by using a fully randomized design. Plants in the first group were used to determine the initial state of immature internodes of all plants to be treated, the second to fifth group plants were assigned respectively for non-submergence treatment (namely as controls) and for complete submergence treatments with the top of plants 2 m, 5 m, and 9 m beneath the water surface in a water-filled concrete reservoir. The plants subjected to complete submergence treatments were suspended at planned water depths, as described in Jing et al. (2022). The design of 2 m, 5 m, and 9 m deep submergence was based on our long-term field observation on the elevational distribution of *A. philoxeroides* in the water level fluctuation zone of the Three Gorges reservoir. Unsubmerged control plants were placed under dark conditions and watered regularly to ensure adequate water supply. All treatments were lasted for six days.

To investigate the effects of submergence depth on plants, the physicochemical status of water body (light, dissolved oxygen, pH, and temperature) in the concrete reservoir were kept constant at any depths. During the experiment, no significant differences in these factors were found between different water depths (**Table 1**).

**Table 1:** Physico-chemical properties of water body in submergence reservoir during the experiment.

Submergence Depth (m)	Dissolved oxygen concentration (mg L^-1^)	Temperature (°C)	pH	PAR (μmol m^-2^ s^-1^)
0	n.a.	26.45 ± 0.07 a	n.a.	0 a
2	8.03 ± 0.08 a	26.43 ± 0.04 a	7.01 ± 0.02 a	0 a
5	7.98 ± 0.09 a	26.45 ± 0.07 a	7.06 ± 0.03 a	0 a
9	7.92 ± 0.05 a	26.45 ± 0.03 a	7.05 ± 0.02 a	0 a

The dissolved oxygen, temperature, photosynthetically active radiation (PAR), and pH of the water body in the concrete reservoir for submergence treatments were checked at different depths twice per day (in the morning and evening) using a multi-parameter water quality analyzer (Hydrolab DS5, Hach, United States) during the experiments (mean ± s. e.; n = 13); n.a. indicates no data. Same lower-case letter indicates no significant difference (p > 0.05) between submergence depths.

### 2.3 Growth measurements

**Figure 1:**
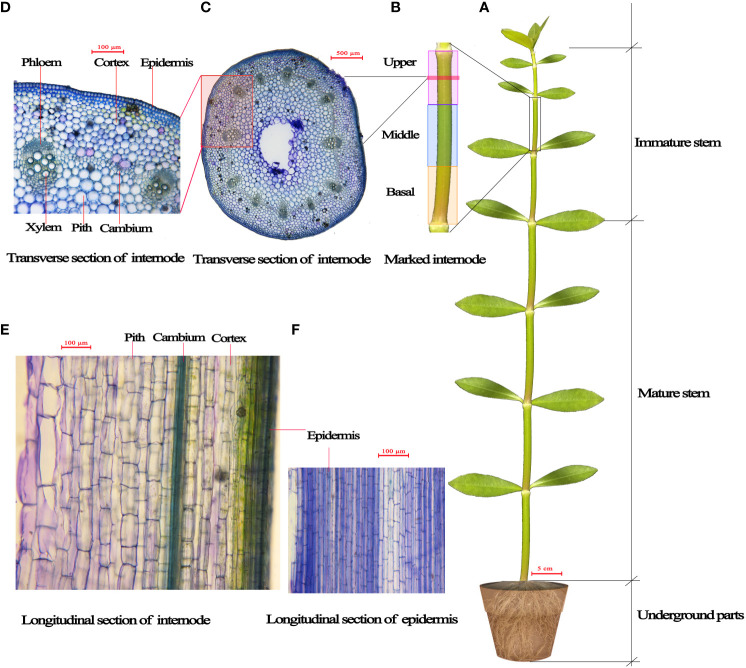
Diagrammatic representation of *Alternanthera philoxeroides*. **(A)** the mature and immature stem internodes; **(B)** the basal, middle and upper part of internode; **(C, D)** the transverse sections of internode; **(E, F)** the longitudinal sections of internode, showing the longitudinal view of epidermis, cortex and pith cells.

Each plant had ~12 stem internodes at the start of treatments. From the stem base upwards, the 1st to 6th internodes were relatively more mature and comprised the mature stem part of the plant, and the 7th to 12th internodes were immature and comprised the immature plant stem part (**Figure 1A**). We distinguished the relatively mature internodes from the immature internodes in each plant before treatments to detect the respective elongation growth of mature and immature stem parts during the treatments. The elongation of stem part (either mature or immature stem part) was defined as the length difference of the stem part before and after treatment.

In order to investigate which part within an immature internode played a larger role in internode elongation growth, we selected an immature internode (the length of the internode was usually between 2.5 and 3.2 cm) from each plant in each of five groups immediately before treatments and divided the internode into three equilong parts (basal, middle, and upper part, as shown in **Figure 1B**) by marking with red polyester threads, measured the lengths of all parts before and after treatments so as to determine the elongation growth of each part.

### 2.4 Longitudinal cell proliferation and enlargement in immature internodes

The longitudinal cell length and cell number of immature internodes before and after submergence treatments were checked by using the first group plants (which were assigned for initial state checking) before submergence and the second to fifth group plants (which were assigned respectively for submergence at water depths of 0 m, 2 m, 5 m, and 9 m) after submergence. The lengths of the basal, middle, and upper parts of the marked internodes (**Figure 1B**) of the first group plants before submergence and of the second to fifth group plants after submergence were measured using a stereomicroscope (SMZ25, Nikon, Japan) with the help of NIS-elements imaging software (version 4.30). Subsequently, a short internode segment about 2~3 mm long was cut out in the middle of the basal, middle, and upper parts of each marked internode to make longitudinal sections (**Figures 1E, F**), the average longitudinal lengths of epidermis, cortex, and pith cells were determined (by respectively measuring the lengths of randomly selected 50 cells) for the basal, middle, and upper parts by staining with 0.5% toluidine blue. The longitudinal number of cells in the basal, middle, and upper parts of marked immature internodes were determined respectively by dividing the length of each part by the cell length.

### 2.5 Data analysis

Elongation difference between mature and immature stem parts of *A. philoxeroides* subjected to each submergence depth were checked by Paired-Samples t test, elongation difference of mature stem parts (or immature stem parts) between submergence depths were checked by one-way ANOVA. ANOVA with Repeated Measures was used to detect the difference respectively in internode length, cell length, or cell number between basal, middle, and upper parts within internodes, One-way ANOVA was used to examine the difference respectively in internode length, cell length, or cell number of basal, middle, or upper parts between different treatments. Logarithm data transformation was performed to equalize variance if necessary. Differences between treatments were detected using the Tukey HSD test, and the significance level was set at *p* = 0.05. The correlations of immature internode length to the cell length and cell number of epidermis, cortex, and pith in the basal, middle, and upper parts of the immature internodes were analyzed using Pearson correlation analysis. All analyses were conducted using SPSS 22 (SPSS Inc., Chicago).

## 3 Results

### 3.1 Elongation of mature and immature stem parts

**Figure 2:**
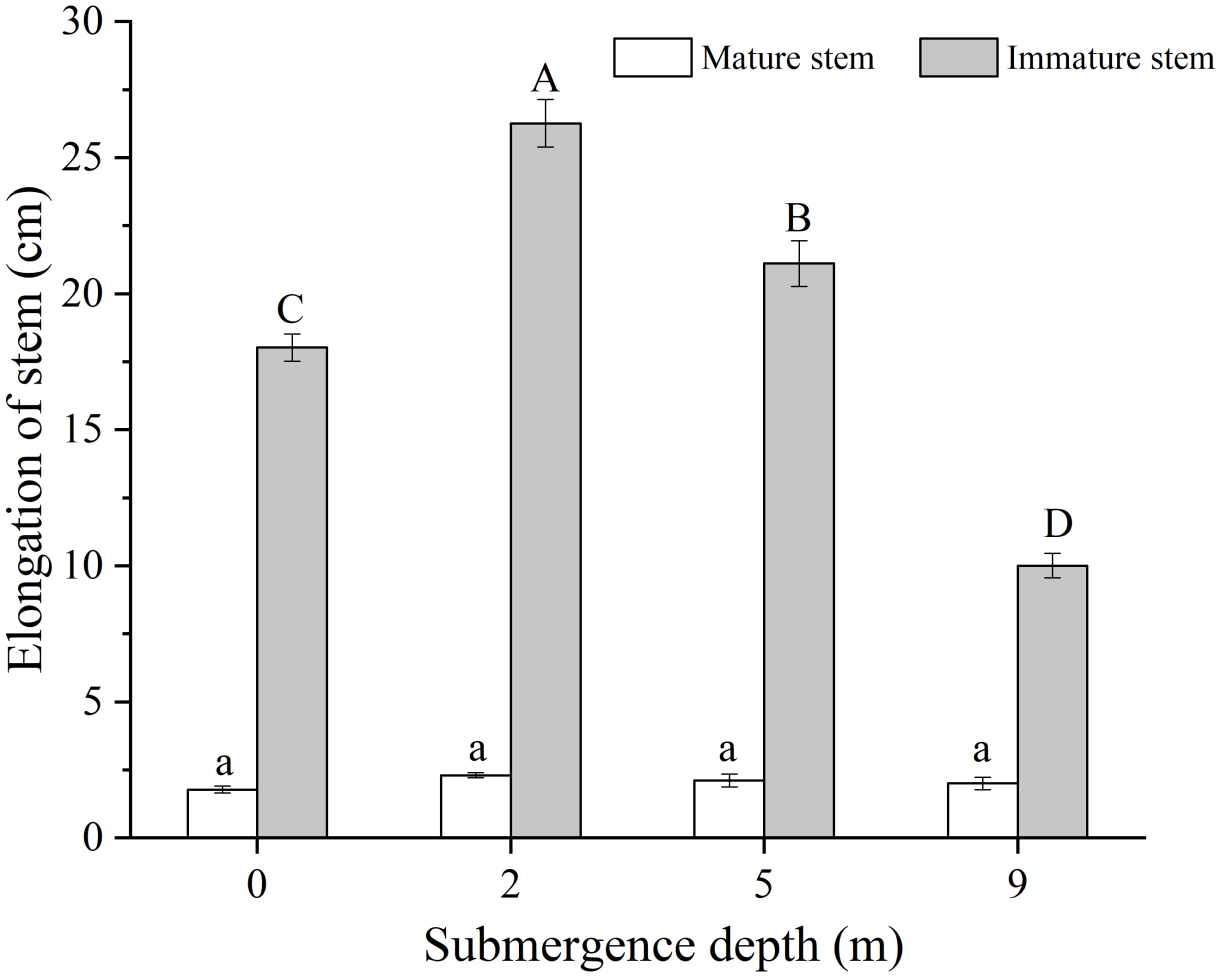
Elongation of the mature and immature stem parts of *Alternanthera philoxeroides* at the end of submergence treatment of different water depths (mean ± S.E; *n* = 20). Same lower-case letters indicate no difference (*p* > 0.05) between treatments in the elongation of mature stem part. Different upper-case letters indicate significant difference (*p* < 0.05) between treatments in the elongation of immature stem parts.

The mature and immature stem parts of *A. philoxeroides* formed under unsubmerged conditions differed in elongation during the experiment, the immature stem parts presented apparent elongation but the mature stem parts only had very slight elongation (**Figure 2**). The final elongation of immature stem parts significantly decreased with increasing submergence depth at the end of treatment, which were 18.02 cm, 26.26 cm, 21.11 cm, and 10.01 cm averagely in plants submerged at depth of 0 m (control), 2 m, 5 m, and 9 m, respectively (**Figure 2**). The immature stem parts in plants submerged at water depth of 2 m and 5 m achieved significantly longer elongation than those of control plants (0 m) but shorter elongation in plants submerged at water depth of 9 m (*p* < 0.05, **Figure 2**). It was shown that immature stem parts made a larger contribution than relatively mature stem parts to the whole stem elongation of *A. philoxeroides* plants when submerged.

### 3.2 Elongation of constituent parts of immature internodes

**Figure 3:**
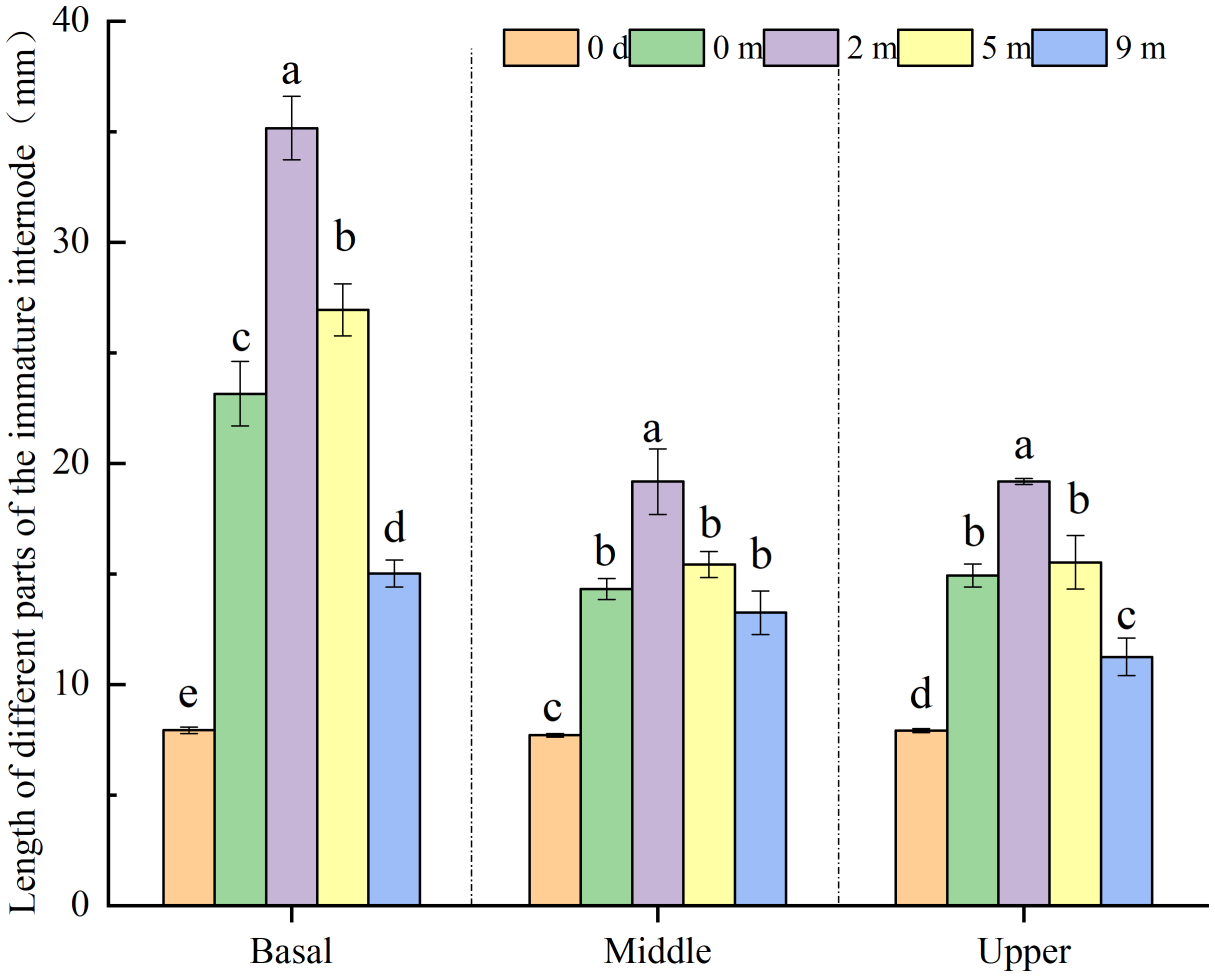
Length of basal, middle and upper part of *Alternanthera philoxeroides* immature internode before (indicated by 0 d, namely 0 day) and at the end of submergence treatment at water depth of 0m, 2m, 5m, and 9m (mean ± S.E; *n* = 12). For either basal, middle, or upper part, different letters indicate significant differences (*p* < 0.05) between treatments.

Each internode of *A. philoxeroides* in this study was seen as being composed of three constituent parts of equal length: basal, middle, and upper part. At the end of submergence treatments, all constituent parts of immature internodes presented significant elongation growth when compared with their initial states at the start of the treatments (**Figure 3**). The basal parts of immature internodes achieved longer length than the middle and upper parts, and there was no significant difference in length between the middle and upper parts (**Figure 3**), which implies that basal parts made the largest contribution to the elongation of internodes among all constituent parts.

The elongation growth of immature *A. philoxeroides* internodes differed significantly in response to submergence depth, all constituent parts of immature internodes including basal, middle, and upper parts gradually decreased their elongation with increasing water depth (**Figure 3**). Plants submerged at water depth of 2 m and 5 m achieved larger lengths than unsubmerged control plants (water depth of 0 m) in all constituent parts of immature internodes, especially in basal parts and for plants submerged at 2 m depth. However, plants submerged at water depth of 9 m exhibited smaller length than unsubmerged control plants in all constituent parts (**Figure 3**).

### 3.3 Cell length in immature internodes

**Figure 4:**
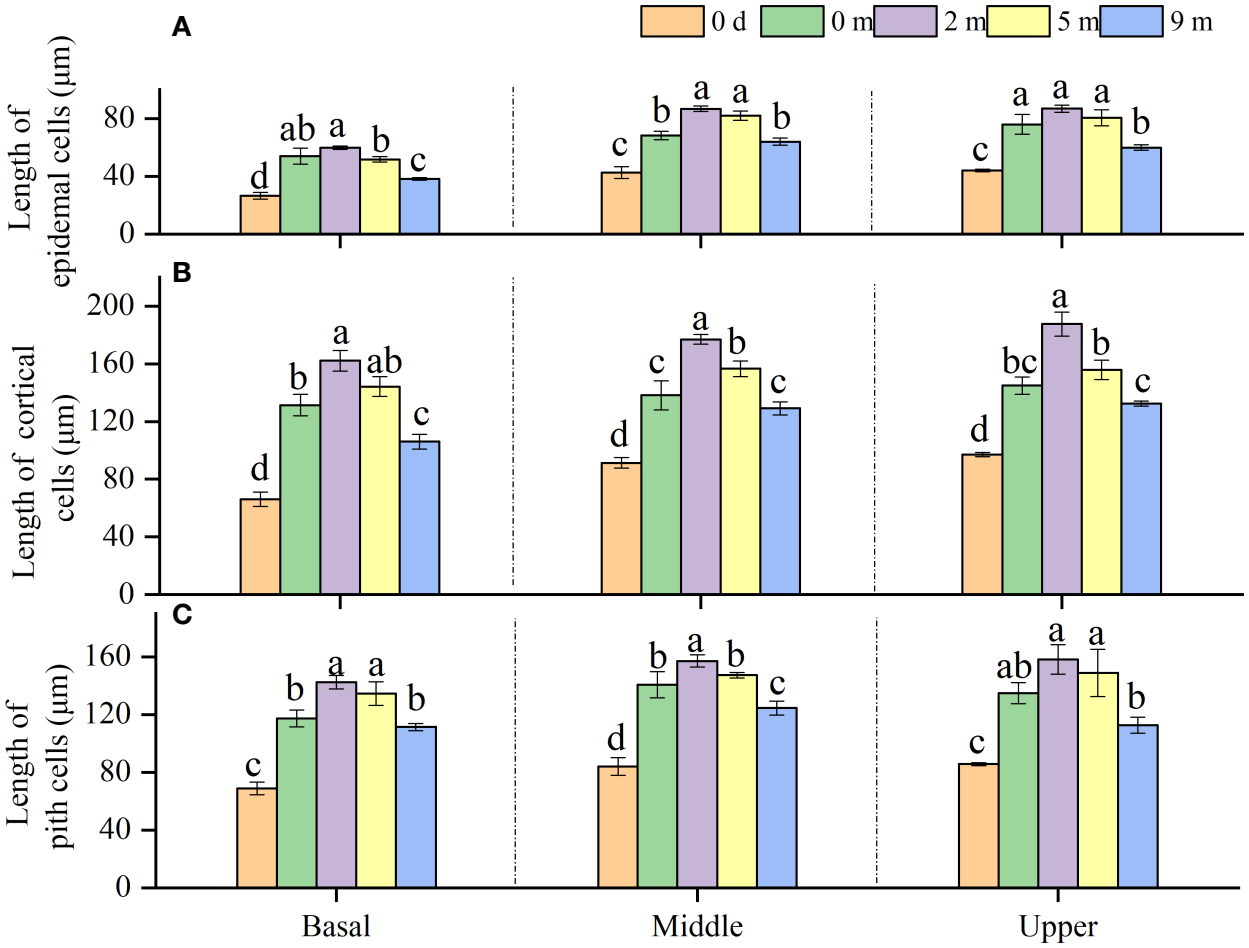
Length of epidermal cells **(A)**, cortical cells **(B)** and pith cells **(C)** in basal, middle, and upper part of *Alternanthera philoxeroides* immature internode before (indicated by 0 d, namely 0 day) and at the end of submergence treatment at water depth of 0m, 2m, 5m, and 9m (mean ± S.E; *n* = 4). For either basal, middle, or upper part, different letters indicate significant differences (*p* < 0.05) between treatments.

It was found that the epidermis cells of *A. philoxeroides* stem internodes were shorter longitudinally than the cortex and pith cells (**Figures 1E, F**, **4**). As can be seen from the plants before submergence treatment and the plants following submergence, longitudinal lengths of epidermis, cortex, and pith cells (**Figures 1C–F**) in the basal parts of immature internodes were shorter than those in the middle and upper parts, and no significant differences in cell lengths between the middle and upper parts were found (**Figure 4**).

Compared with the cells in the basal, middle, and upper parts of immature internodes at the start of treatment (0 d), the cells of any sorts (epidermis, cortex, and pith) in the basal, middle, and upper parts increased their longitudinal length after treatments (**Figure 4**). The lengths of cells of any sorts in the basal, middle, and upper parts of the internodes decreased with increasing submergence depth (**Figure 4**). All cells in any internode parts presented the longest lengths in plants submerged at 2 m depth. Comparatively, at the end of the treatment, the plants submerged at 2 m and 5 m depth had longer epidermis, cortex, and pith cells in all internode parts than unsubmerged control plants, but the plants submerged at 9 m depth showed shorter cells than unsubmerged control plants (**Figure 4**).

### 3.4 Cell number in immature internodes

**Figure 5:**
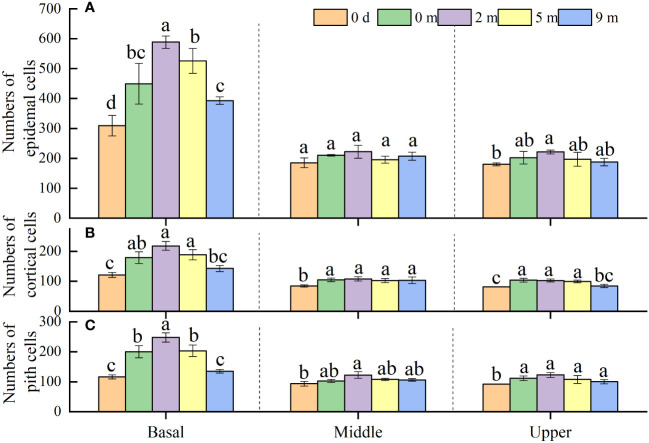
Number of epidermal cells **(A)**, cortical cells **(B)** and pith cells **(C)** in basal, middle, and upper part of *Alternanthera philoxeroides* immature internode before (indicated by 0 d, namely 0 day) and at the end of submergence treatment at water depth of 0m, 2m, 5m, and 9m (mean ± S.E; *n* = 4). For either basal, middle, or upper part, different letters indicate significant differences (*p* < 0.05) between treatments.

Under natural conditions, the basal parts of immature internodes had a larger number of cells than the middle and upper parts, and no significant difference in cell number was shown between the middle and upper parts (**Figure 5**). At the end of treatment, the numbers of epidermis, cortex and pith cells in the basal parts of immature internodes of all submerged plants increased (except the number of cortex and pith cells in plants submerged at 9 m depth) when compared to those before the treatment, but the numbers of all sorts of cells at the middle and upper parts showed no increase at all (**Figure 5**). As to the basal parts of immature internodes, the number of all sorts of cells therein decreased gradually with increasing submergence depth at the end of treatments; and generally, plants submerged at 2 m and 5 m depth had larger number of cells but plants submerged at 9 m depth had smaller number of cells than unsubmerged control plants (**Figure 5**).

### 3.5 Cell proliferation and enlargement in relation to internode elongation

**Figure 6:**
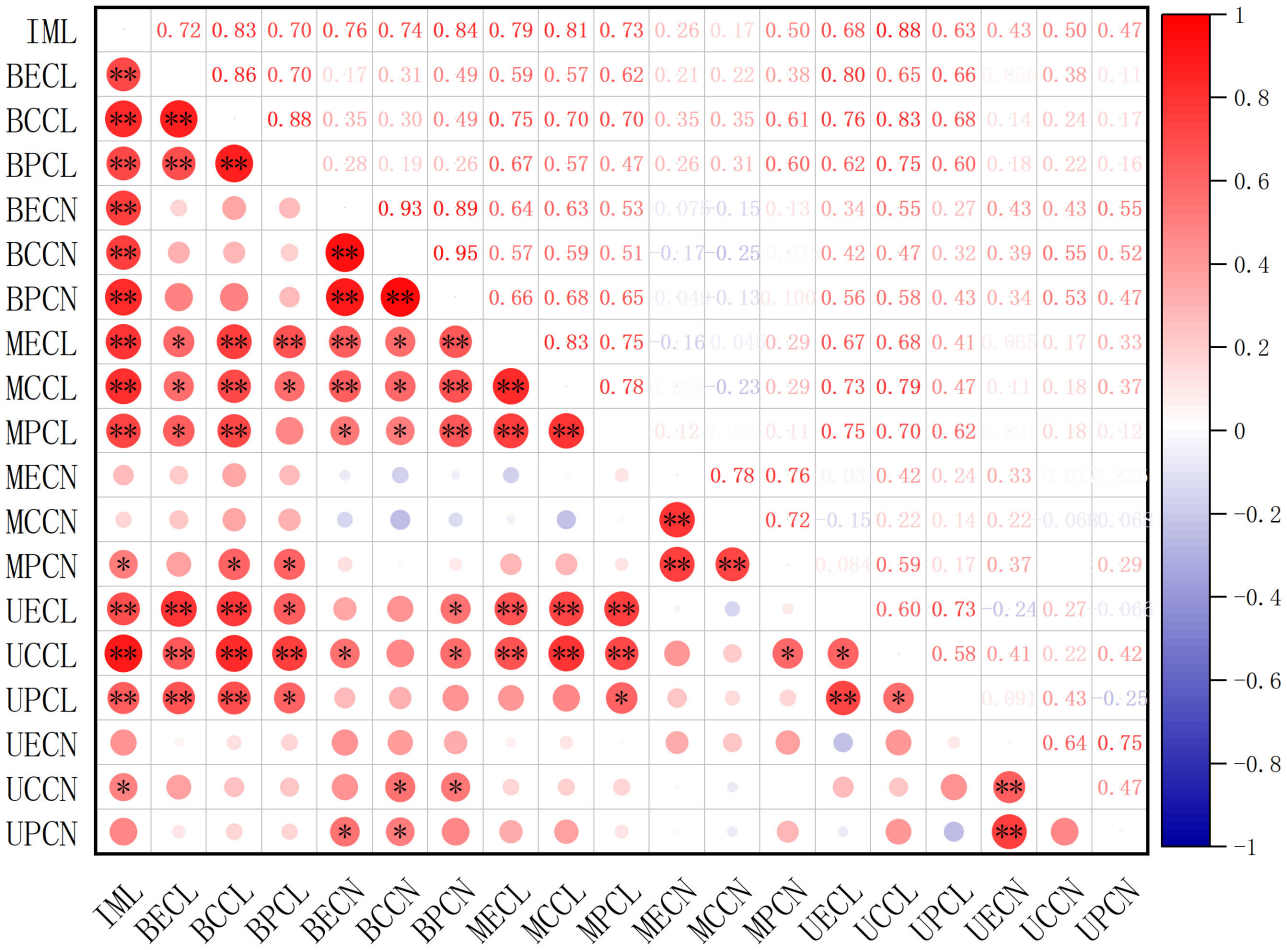
The correlation of immature internode length of *Alternanthera philoxeroides* to the cell length and cell number of epidermis, cortex, pith in the basal, middle, and upper part of the immature internode at the end of treatment. Red and blue circle indicates respectively positive and negative correlation between variables; the larger the circle area, the higher the correlation. * and ** indicates respectively significant correlation at *p* < 0.05 and highly significant correlation at *p* < 0.01. IML refers to immature internode length; BECL, BCCL, BPCL, MECL, MCCL, MPCL, UECL, UCCL, UPCL refer to epidermis cell length, cortex cell length, pith cell length of internode basal part, epidermis cell length, cortex cell length, pith cell length of internode middle part, epidermis cell length, cortex cell length, pith cell length of internode upper part, respectively; BECN, BCCN, BPCN, MECN, MCCN, MPCN, UECN, UCCN, UPCN refer to epidermis cell number, cortex cell number, pith cell number of internode basal part, epidermis cell number, cortex cell number, pith cell number of internode middle part, epidermis cell number, cortex cell number, pith cell number of internode upper part, respectively.

The contributions of cell proliferation and enlargement to the elongation of immature internodes in *A. philoxeroides* upon submergence were analyzed using Pearson correlation analysis (**Figure 6**). The elongation of immature internodes significantly correlated with the length of cells of any sort (epidermis, cortex, or pith cells) in any part (basal, middle, or upper part) of the internodes, which means that all sorts of cells in all parts made contributions to the internode elongation by cell enlargement. However, as to the correlation of internode elongation to cell proliferation, only the basal parts showed marked correlation of cell number to the internode elongation, both the middle and upper parts did not present strong correlations, which indicated that only cells in the basal part among all internode parts contributed to the internode elongation through cell proliferation.

## 4 Discussion

It has been reported that stems of different maturity contributed differently to plant growth, and the variation in stem elongation of *A. philoxeroides* subjected to submergence of different intensities was related to the performance of young stems (Rankenberg et al., 2021; Jing et al., 2022), but so far the intrinsic anatomical mechanisms therein are still scarce. The objective of the present study was to elucidate the morphological and anatomical mechanisms underlying the different elongation growth of young stems in completely submerged *A. philoxeroides* at different water depths, and to provide insights into the intra-internodal differential responsiveness in elongation growth to submergence depths as well as the roles of cell enlargement and proliferation played in the stem elongation.

### 4.1 Internode elongation as submergence depth changes

Plants respond differently to submergence (van Veen et al., 2014). Some plants employing escape strategies maximize leaf or stem elongation to escape from flooded environments and receive more light, oxygen, carbon dioxide, etc., for photosynthesis to prolong plant survival underwater (Panda and Barik, 2021; Wang and Komatsu, 2022). For example, *Chloris gayana* Kunth. cv. Fine Cut (a submergence-tolerant cultivar) can rapidly extend its leaves above the water surface and increase its biomass significantly after being submerged (Striker et al., 2017). However, some plants employ quiescence strategy by stopping growing or growing slowly to conserve energy for survival (Bailey-Serres and Voesenek, 2008). Pervasively, plants adopt either escape or quiescence strategy during submergence, but it was reported that a special species, *Alternanthera philoxeroides*, behaved as an escape strategist through stem elongation when submerged at 2 m depth and gradually shifted its strategy to quiescence as the depth of submergence increased (Jing et al., 2022). In the present study, we found that it was the immature stem internodes of completely submerged *A. philoxeroides* displayed reducing elongation with increasing water depth, mature internodes did not exhibit this pattern although they obtained a little elongation (**Figure 2**). Moreover, the immature internodes showed intra-internodal variation in elongation among their basal, middle, and upper parts, and the variation was affected by submergence depth. The basal parts achieved much longer elongation than the middle and upper parts at 2 m water depth, but this elongation difference faded away when the water depth increased gradually to 9 m (**Figure 3**). In our previous study (Jing et al., 2022), we found that *A. philoxeroides* plants submerged under 9 m still held good carbohydrates reserve as compared to shallowly submerged plants, which enables the plants stressed by 9 m deep submergence to have sufficient energy for post-submergence growth. Furthermore, in the field, we always see in the drawdown zone of the Three Gorges reservoir in China, which has a large fluctuation of water level (more than 10 m), plants of *A. philoxeroides* growing at the lower elevations of the zone display good growth as plants at higher elevations of the zone when the zone de-submerges every year. Given these facts, we think that the growth retardation under 9 m is more quiescent than being impaired.

In the case of deepwater rice (*Ovyza sativa* L.), the growth of meristematic, elongation, and maturation zones of young internodes differed under partial submergence (Sauter, 2000). Deepwater rice elongation is determined in large part by intercalary meristems at the base of internodes (Sauter, 2000). Submergence shortens the cell division cycle of the meristematic cells, which in turn increases the number of cells (Lorbiecke and Sauter, 1998); moreover, the action of gibberellin increases cell length. These two processes together promote stem elongation (Sauter and Kende, 1992). Deepwater rice is a graminaceous monocot, intercalary meristem is quite common in this plant which results in longitudinal elongation growth pattern (Kende et al., 1998). However, the plant *A. philoxeroides* investigated in our study is dicotyledonous; to date, very few reports on whether *A. philoxeroides* has intercalary meristem is available (Duan et al., 2019). The water depth of submergence had a strong effect on the elongation of basal parts in immature internodes of *A. philoxeroides* in our study, if the elongation of these internodes was closely related to the increase of cell number in the basal parts of the internodes, it would indicate reasonably that the internodes possess intercalary meristems at the base.

### 4.2 Internode elongation and cell contribution

There is a broad consensus that internode elongation can be attributed to increase in cell number and/or cell length (Guttridge and Thompson, 1959). In the present study, we found cells of epidermis, cortex, and pith in the internodes of *A. philoxeroides* differed in size (**Figure 4**), which is consistent with other studies (Crang et al., 2018). Interestingly, it is found in the present study that cells of the same type varied in size in different parts within an internode (**Figure 4**). Cells in the basal parts of immature internodes were shorter and numerous, whereas those in the middle and upper parts were relatively longer and smaller in number (**Figures 4**, **5**). These phenomena were similar to those in young internodes of rice (Kende et al., 1998) and maize (Wang et al., 2021), whose longitudinal internode growth is known driven by the activity of intercalary meristems. In our study the basal parts of *A. philoxeroides* internodes increased their cell number after a period of growth under unsubmerged conditions but the middle and upper parts did not (**Figure 5**), this indicates that internode elongation of *A. philoxeroides* was linked to the activity of intercalary meristems at the base of internodes like what the young internodes of rice and maize do.

Based on the variations in cell length and cell number among internode parts of *A. philoxeroides* subjected to submergence of different water depths, it was found that the varied stem elongation of plants submerged at different depths was caused by disparate cell performance. In comparison with unsubmerged plants, the cell enlargement and cell proliferation were intensified in plants submerged at depth of 2 m but suppressed in plants submerged at depth of 9 m (**Figures 4**, **5**), which resulted in an enhanced stem elongation in the former but retarded stem elongation in the latter (**Figures 2**, **3**). In an analysis on the correlation of internode elongation to cell performance by taking the length and number of all sorts of cells in all three internode parts into consideration, it was shown that the internode elongation was mainly due to the cell proliferation and enlargement at basal parts and partly to the cell enlargement at middle and upper parts (**Figure 6**), which implies that the cells of the basal parts made the largest contribution to internode elongation. In contrast to that cell enlargement and cell number increase both involved in the elongation of the basal parts of *A. philoxeroides*, the elongation of the middle and upper parts was powered only by cell length increment (**Figures 3** **–** **5**).

### 4.3 Mechanisms underlying water depth effect need to be uncovered

It was demonstrated in previous studies that a certain level of pressure can affect plant growth (Mota et al., 2013), and exposure of plants to mechanical forces dramatically resulted in morphological and microstructural alterations in growth (Braam, 2005; Zhou et al., 2007; Brenya et al., 2022). The orientation of cellulose microfibrils (CMFs) was found to be a determinant in cell growth, faster elongation of internodes occurred when CMFs were oriented transversely and slower elongation of internodes took place when CMFs were oriented obliquely or longitudinally (Sauter et al., 1993). Sauter et al. (1993) observed that GA had a significant effect on CMFs orientation in deepwater rice epidermis cells, and submergence triggered the degradation of abscisic acid, which is a GA antagonist, thereby enhancing the GA response and promoting shoot elongation (Sauter and Kende, 1992; Ayano et al., 2014). Therefore, it is possible that the increase in cell number and cell length of *A. philoxeroides* submerged at 2 m water depth in our study was related to the biosynthesis of phytohormones and CMFs orientation, which promoted accelerated internode elongation growth under complete submergence.

However, it was revealed that as the submergence depth increased, the plasma membrane permeability and the malondialdehyde content of live stems increased significantly (Lei et al., 2014). In the process of GA biosynthesis, the second phase of the biosynthesis occurs in endoplasmic reticulum (Taiz and Zeiger, 2010). If increase in hydrostatic pressure on plants caused by deep submergence leads to endoplasmic reticulum instability or damage, it would very likely disturb GA biosynthesis and hinder subsequent cell division and elongation. However, it needs to be investigated further whether an increase in hydrostatic pressure would cause cell membrane instability and affect endogenous phytohormone synthesis, which in turn affects plant growth.

## Data availability statement

The raw data supporting the conclusions of this article will be made available by the authors, without undue reservation.

## Author contributions

SJ: Data curation, Formal analysis, Investigation, Writing – original draft. XR: Writing – original draft, Investigation. FL: Formal analysis, Investigation, Writing – original draft. HN: Writing – original draft. QA: Investigation, Methodology, Writing – original draft. BW: Investigation, Writing – original draft. BZ: Investigation, Methodology, Writing – original draft, Conceptualization, Formal analysis, Funding acquisition, Project administration, Resources, Supervision, Validation, Visualization, Writing – review & editing. XZ: Conceptualization, Funding acquisition, Methodology, Project administration, Writing – original draft, Formal analysis, Writing – review & editing.
